# The Diagnostic Accuracy of [^18^F]Fluorocholine PET/CT for the Preoperative Localization of Hyperfunctioning Parathyroid Glands

**DOI:** 10.3390/diagnostics16182950

**Published:** 2026-09-12

**Authors:** Chiara Lauri, Giuseppe Campagna, Lorenzo Chiurchioni, Luciano Carideo, Michela Varani, Valeria Bentivoglio, Anna Giorgio, Francesco D’Angelo, Alberto Signore

**Affiliations:** 1 Nuclear Medicine Unit, Department of Medical-Surgical Sciences and of Translational Medicine, Faculty of Medicine and Psychology, “Sapienza” University, 00189 Rome, Italy; giuseppe.campagna@uniroma1.it (G.C.); lorenzo.chiurchioni@gmail.com (L.C.); lcarideo@ospedalesantandrea.it (L.C.); michela.varani@uniroma1.it (M.V.); valeria.bentivoglio@uniroma1.it (V.B.); anna.giorgio@phd.unipd.it (A.G.); alberto.signore@uniroma1.it (A.S.); 2 Thyroid and Parathyroid Surgery Unit, Department of Neurosciences, Mental Health and Sensory Organs, Faculty of Medicine and Psychology, “Sapienza” University, 00189 Rome, Italy; francesco.dangelo@uniroma1.it

**Keywords:** parathyroid adenoma, parathyroid hyperplasia, parathyroid carcinoma, [^18^F]fluorocholine PET/CT, [^99m^Tc]Tc-MIBI/[^99m^Tc]TcO_4_, US

## Abstract

**Background/Objectives:** The purpose of this study was to compare [^18^F]Fluorocholine (FCH) PET/CT, ultrasound (US) and dual-phase scintigraphy with [^99m^Tc]Tc-methoxyisobutylisonitrile (MIBI) and [^99m^Tc]Tc-pertechnetate (TcO_4_-) ([^99m^Tc]Tc-MIBI/[^99m^Tc]TcO_4_) in the preoperative detection of hyperfunctioning parathyroid glands. Secondary aims were to determine the optimal scanning time of [^18^F]FCH PET/CT while investigating the correlation between [^18^F]FCH PET/CT parameters and PTH, calcium levels, lesions size, histopathology, and immunohistochemical analysis. **Methods:** Patients with primary hyperparathyroidism who underwent US, [^99m^Tc]Tc-MIBI/[^99m^Tc]TcO_4_ scintigraphy, [^18^F]FCH PET/CT and parathyroid surgery were retrospectively evaluated. The following PET parameters were calculated: SUVmax and SUVmean, parathyroid retention index (RI) and lesion contrast (LC). Lesions were classified as parathyroid adenomas (PA), hyperplastic parathyroid glands (HP) or parathyroid carcinoma (PC) according to histology. **Results:** Forty-nine patients were evaluated and 55 lesions were removed at surgery (8 normal glands, 33 PAs, 12 HP glands, 2 PCs). In a lesion-based analysis, the sensitivity and specificity were 68.9% and 28.6% for US, 65.7% and 40.0% for [^99m^Tc]Tc-MIBI/[^99m^Tc]TcO_4_, 97.8% and 14.3% for early-phase [^18^F]FCH PET/CT and 95.7% and 12.5% for late-phase [^18^F]FCH PET/CT. The sensitivity of both early and late [^18^F]FCH PET/CT acquisitions was statistically higher than that of US and [^99m^Tc]Tc-MIBI/[^99m^Tc]TcO_4_ scintigraphy. SUVs were statistically higher in PA than in HP, and RI was significantly lower in HP compared with PC, although this difference was based only on two PC cases. Therefore, this result must be considered preliminary and interpreted with caution. **Conclusions:** [^18^F]FCH PET/CT performed better than US and [^99m^Tc]Tc-MIBI/[^99m^Tc]TcO_4_ scan for the detection of hyperfunctioning lesions. The most favorable time point to assess parathyroid lesions appears to be 60 min after injection. However, due to the possible rapid washout of some lesions, a supplementary early acquisition is advisable. SUVmax, SUVmean and RI may predict the histological nature of a parathyroid lesion, but larger studies are needed to confirm these findings.

## 1. Introduction

Primary hyperparathyroidism (pHPT) is the third most common endocrine disorder and is characterized by autonomous overproduction of parathyroid hormone (PTH), resulting in renal, cardiovascular, and bone complications due to hypercalcemia. In approximately 80–85% of cases, pHPT is caused by a solitary benign adenoma, but pHPT can also be due to multiple adenomas (15 to 20%), diffuse or nodular hyperplasia (<15%), while parathyroid carcinoma (PC) accounts for less than 1% of all diagnoses [1,2]. Surgical removal of the hyperfunctioning gland(s) provides a definitive cure and is recommended for all symptomatic patients and selected asymptomatic patients according to current international guidelines [3,4]. Bilateral neck exploration has long been the standard surgical approach. Currently, minimally invasive parathyroidectomy is preferred in patients with single-gland disease and no concomitant thyroid disease or family history of multiple endocrine neoplasia syndrome (MEN) [4], allowing shorter surgical times, reduced surgical invasiveness, and faster patient recovery. Therefore, imaging has become an integral part of the diagnostic work-up to provide precise preoperative localization rather than to establish the diagnosis, which remains biochemical.

Cervical ultrasonography (US) and dual-phase dual-tracer [^99m^Tc]Tc-methoxyisobutylisonitrile (MIBI)/[^99m^Tc]Tc-pertechnetate (TcO_4_) ([^99m^Tc]Tc-MIBI/[^99m^Tc]TcO_4_) scintigraphy, preferably integrated with single-photon emission tomography/computed tomography (SPECT/CT), are the conventional first-line imaging modalities for preoperative localization of parathyroid glands [5,6]. Recent advances in US technology, including microvascular flow imaging, contrast-enhanced ultrasound (CEUS), and elastography, may further expand the information obtainable from conventional ultrasound by providing complementary assessment of microvascularity, perfusion dynamics, and tissue stiffness.

In cases of negative or doubtful results with persisting clinical suspicion, second-line imaging may be performed with four-dimensional CT (4D-CT) or positron emission tomography (PET) combined with CT (PET/CT) or with magnetic resonance imaging (PET/MRI) [7,8,9,10]. Radiolabelled choline analogs, mainly [^18^F]fluorocholine ([^18^F]FCH), due to their technical and physical advantages compared with [^11^C]CH, are nowadays commonly indicated for parathyroid imaging [5,6]. Increased uptake is thought to reflect the enhanced phospholipid membrane synthesis and increased choline kinase activity due to PTH secretion [11,12]. Evidence has accumulated over the last 10 years, and numerous prospective and retrospective studies and meta-analyses consistently report sensitivities exceeding 90–95%, demonstrating superior diagnostic performance compared with conventional first-line modalities [12,13,14,15,16,17,18].

In particular, the multicenter randomized APACH2 trial recently demonstrated that [^18^F]FCH PET/CT is an effective alternative to conventional scintigraphy as first-line functional imaging in patients with pHPT, thus supporting its integration into future diagnostic algorithms [19,20,21,22]. Cost-effectiveness studies recently concluded that performing a [^18^F]FCH PET/CT as an alternative to a MIBI scan allows a larger proportion of patients to be directly referred to effective minimally invasive parathyroidectomy with minimal additional cost [20,21,22].

Due to the accumulated evidence, many endocrine surgery and nuclear medicine centers have progressively adopted [^18^F]FCH PET/CT earlier in the diagnostic work-up of pHPT, and an update of current imaging recommendations is expected in the near future [15].

Therefore, the main focus is no longer to demonstrate that [^18^F]FCH PET/CT performs better than conventional imaging tests, but rather to address how it could be integrated into the preoperative work-up of parathyroid lesions.

Nevertheless, several technical issues remain unresolved. First, there is still no unanimous consensus regarding the optimal acquisition time since the protocols for [^18^F]FCH PET/CT vary widely across studies. The dual-phase examination protocol usually consists of an early acquisition at 5 to 10 min post-injection (p.i.), allowing the detection of parathyroid lesions with a rapid washout, and a late acquisition at one hour p.i., which has been shown to provide a higher lesion-to-background and lesion-to-thyroid ratio. Moreover, the potential added role of semiquantitative analysis beyond lesion detection and its relationship with tumor histology has not been extensively investigated.

The aims of this study were to confirm the higher diagnostic performance of [^18^F]FCH PET/CT in the preoperative detection of hyperfunctioning parathyroid glands in comparison with US and [^99m^Tc]Tc-MIBI/[^99m^Tc]TcO_4_ scintigraphy, to compare the semiquantitative PET parameters of early and late acquisitions, to determine the optimal scanning time of [^18^F]FCH PET/CT, and to investigate the correlation between [^18^F]FCH PET/CT parameters and PTH and calcium levels, the size of the parathyroid lesions, and histopathological markers.

## 2. Materials and Methods

### 2.1. Patient Selection

In this single-center retrospective study, we retrospectively recruited patients (>18 years old) with biochemically diagnosed pHPT (high PTH and high calcium levels), who were candidates for surgery and underwent first-line imaging procedures (US and [^99m^Tc]Tc-MIBI/[^99m^Tc]TcO_4_ scintigraphy) and dual-phase [^18^F]FCH PET/CT at our institution between November 2014 and May 2022. PTH and calcium levels at the time of [^18^F]FCH PET/CT and the final diagnosis achieved by histology were collected. The exclusion criteria were patients who did not undergo surgical treatment, patients with secondary or tertiary hyperparathyroidism, patients with persistent or recurrent pHPT after surgery, and patients with a known history of malignant and/or inflammatory disease of the head and neck region.

### 2.2. Cervical Ultrasonography

Cervical US using a high-definition linear probe of 13 MHz and color Doppler was performed by experienced radiologists as the first imaging examination for all patients. Results were considered positive in the case of a hypoechoic parathyroid lesion measuring ≥1 cm with an oval or bean shape. Results were considered negative in the absence of a suspicious enlarged parathyroid gland. Results were considered equivocal if findings could not confidently be referred to as parathyroid tissue. For each positive result, the location (right superior/inferior and left superior/inferior in the thyroid bed) and the three diameters of each lesion were recorded.

### 2.3. [^99m^Tc]Tc-MIBI/[^99m^Tc]TcO_4_ Scintigraphy

All patients followed a dual-tracer, dual-phase [^99m^Tc]Tc-MIBI/[^99m^Tc]TcO_4_ protocol.

**Figure 1:**
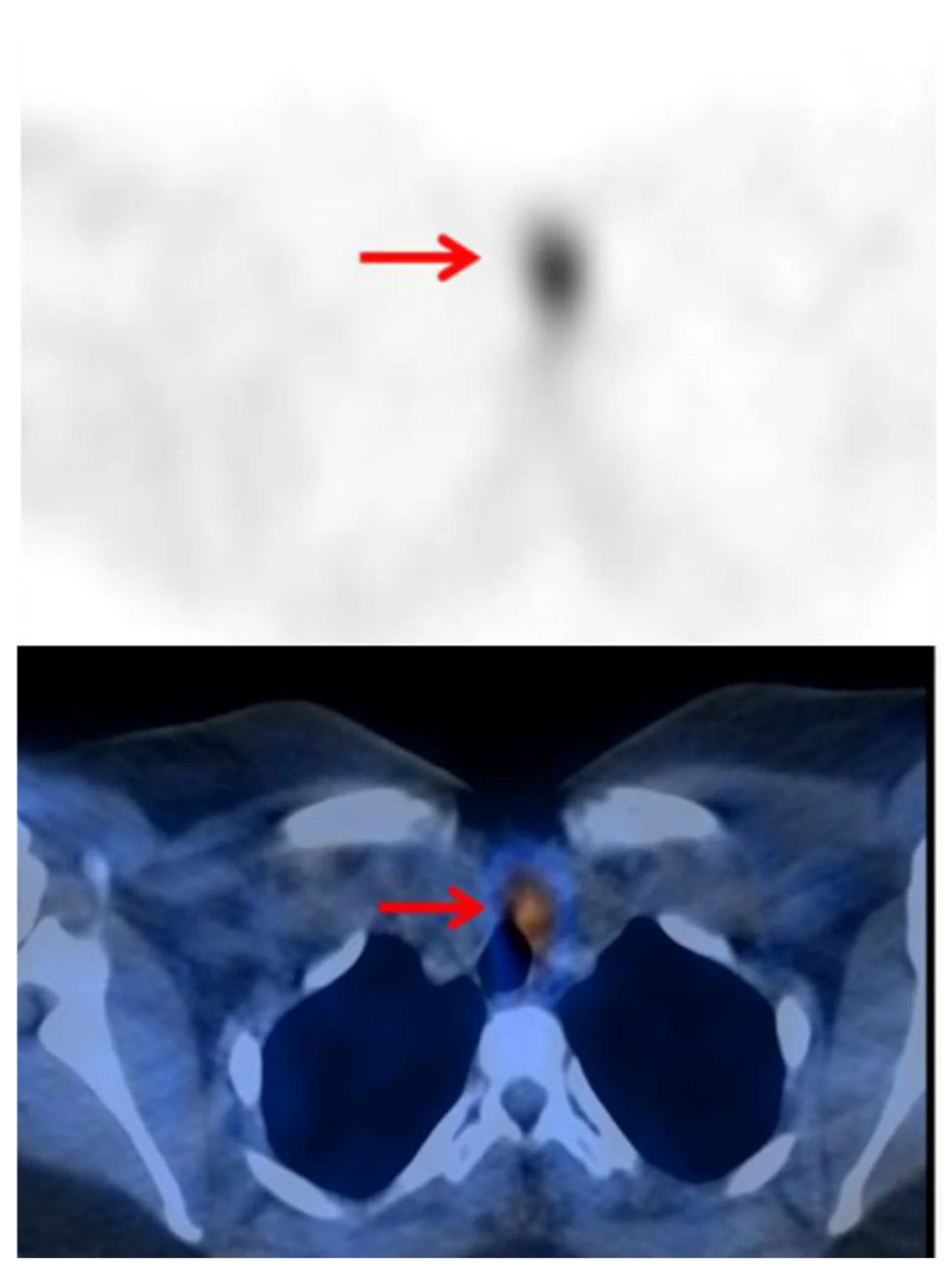
SPECT (**upper panel**) and fused SPECT/CT (**lower panel**) of [^99m^Tc]Tc-MIBI in a patient with left PA (red arrow).

A dose of 740 MBq of [99mTc]Tc-MIBI was intravenously injected, and anterior planar images of the neck and mediastinum were obtained at 10, 60, and 120 min. All acquisitions were performed using a large field-of-view gamma camera (Philips Sky-light, Philips Medical Solution, Best, The Netherlands) fitted with a low-energy high-resolution collimator, a matrix size of 256 × 256, a 20% energy window centered at the 140 keV (^99m^Tc) photon peak, and a digital zoom of 2.19. The first [^99m^Tc]Tc-MIBI image was acquired for 600 s, and the following images were acquired with times corrected for isotope decay (673 s and 756 s, respectively). Planar images were followed by a single-photon emission tomography (SPECT) acquisition at 60 min after [^99m^Tc]Tc-MIBI injection. The SPECT acquisition comprised 64 projections of 35 s each. After the last planar [^99m^Tc]Tc-MIBI acquisition (120 min), [^99m^Tc]TcO_4_ (150 MBq) was injected, and images were acquired after 15–20 min, without moving the head of the patient [5,6] (Figure 1).

Images were interpreted by an experienced nuclear medicine physician.

At the end of the examination, early and delayed images were inspected visually before and after subtraction. Abnormal focal uptake that was sustained on delayed imaging or persisted after subtraction, close to the thyroid bed or at any potential site of ectopic uptake, was considered positive. The location (right superior/inferior and left superior/inferior in the thyroid bed or ectopic location) and the number of suspicious foci were recorded.

### 2.4. [^18^F]FCH PET/CT

PET and CT images were acquired using a PET/CT scanner (Biograph Horizon, Siemens Medical Solutions, Malvern, PA, USA). PET images were obtained at 10 min (early phase) and 60 min (late phase) after injection of 185 MBq of [^18^F]FCH. The field of view included the entire neck and the upper mediastinum. Acquisition time was set to 2.5 min per bed position. A low-dose CT scan covering the same field of view was acquired for attenuation correction and topographic localization. The CT parameters were 140 kV, 70 mA, 0.5 s per rotation, a pitch of 6:1, with a slice thickness of 5 mm. Integrated PET/CT images were automatically obtained by co-registration and fusion of the CT and PET images using a commercially available image registration software tool (SyngoVia, Siemens Healthineers, Erlangen, Germany).

[^18^F]FCH PET/CT images were assessed by two nuclear medicine physicians. A well-defined focal uptake higher than the adjacent background activity, located in the typical neck location and corresponding to a nodular lesion on CT, was considered positive. A volume of interest (VOI) was manually delineated on each suspicious focal uptake and on the adjacent thyroid tissue at both time points. The following PET parameters were analyzed: maximum and mean standardized uptake values (SUVmax and SUVmean), retention index (RI), and lesion contrast (LC). SUVmax represents the voxel with the highest uptake. SUVmean is the mean uptake within the VOI. SUVmean values were used to calculate RI and LC, as applied in the study by Rep et al. [23].

The RI of a lesion/tissue represents the percentage variation in the SUVmean between the early and late phases. RI was calculated for each parathyroid lesion using the following formula:

$$\mathrm{RI}\;=\frac{{SUV}_{mean\;late}-{SUV}_{mean\;early}}{{SUV}_{mean\;early}}\times 100$$

LC refers to the difference in signal intensity between the parathyroid lesion (P) and thyroid tissue (T). Accumulation of [^18^F]FCH is usually higher in the parathyroid lesion than in the thyroid tissue, but in some cases it may be lower, thus resulting in a negative LC value. LC was calculated in both early and late phases by comparing SUVmean for the parathyroid gland (SUV^P^mean) and SUVmean for the thyroid gland (SUV^T^mean) using the following formula:

$$LC=\frac{{SUV}_{mean}^{p}-{SUV}_{mean}^{T}}{{SUV}_{mean}^{T}}\times 100$$

### 2.5. Histological and Immunohistochemical Analysis

Histological analysis was performed on all surgically removed parathyroid glands as the standard of reference. According to imaging tests, surgical approaches included parathyroidectomy of the overactive glands in cases of positive examination, or cervical exploration with parathyroid removal according to the surgeon’s experience in cases of negative imaging. Samples were identified as parathyroid adenomas (PA), hyperplastic parathyroid glands (HP), parathyroid carcinoma (PC), healthy parathyroid, or non-parathyroid tissues. Immunohistochemical analysis was performed in a subgroup of patients, including the following markers: Ki-67, Gal3, BCL2, and p53. High expression values for Ki-67, Gal3, and BCL2 were considered to be ≥5%, ≥30%, and ≥30% of the normal cut-off, respectively. Positivity for p53 was defined when any cell demonstrated nuclear p53 staining.

### 2.6. Imaging-Pathology Correlation

To define the imaging-pathology correspondence of a lesion, each parathyroid lesion detected on imaging was compared with the corresponding surgically removed lesion on the same side of the thyroid bed or in an ectopic position.

True-positive (TP) findings were defined as an abnormal parathyroid gland on the same side of the thyroid bed or in an ectopic position, which was further confirmed by histological analysis of the corresponding specimen. An abnormal parathyroid gland with no further confirmation on histological analysis was considered a false-positive (FP). The absence of an abnormal image or focus at a site with corresponding positive histological analysis was considered a false negative (FN). The absence of an abnormal image or focus at a site with corresponding negative histological analysis was considered a true negative (TN).

### 2.7. Statistical Analysis

Continuous variables were expressed as mean ± standard deviation (SD), and categorical variables were presented as frequency and percentage.

Comparisons between sensitivity (Se), specificity (Sp), positive predictive value (PPV), negative predictive value (NPV), and accuracy for each imaging modality were tested by a z-test for the equality of two proportions. Se, Sp, PPV, NPV, and accuracy were calculated using SAS v. 9.4.

A generalized linear mixed model (GLIMMIX) was performed to evaluate diagnostic performance of neck US, [^99m^Tc]Tc-MIBI/[^99m^Tc]TcO_4_ scan and [^18^F]FCH PET/CT and quantitative parameters.

Normality of the continuous variables was tested using the Shapiro–Wilk test and by checking the Q–Q plot.

Correlations between PET parameters and PTH levels, calcium levels, and lesion size were obtained by Kendall’s tau-b correlation.

The Brunner–Munzel test or Student’s *t*-test was used to compare the continuous variables according to lesion size (≤1 cm^3^ vs. >1 cm^3^).

The trend of immunohistochemistry markers according to the different hyperfunctioning lesions was evaluated by the Cochran–Armitage trend test.

Statistical analysis was performed using SAS v.9.4 TS Level 1M8, and JMP PRO v. 17.1 (Institute Inc., Cary, NC, USA). A *p*-value <0.05 was considered statistically significant.

## 3. Results

### 3.1. Patient Population

Forty-nine patients were included, and 55 parathyroid lesions were surgically removed. Mean calcium levels, PTH levels, and lesion sizes are shown in Table 1. All patients showed normal creatinine and vitamin D values.

US was performed in 46 patients and identified 52 suspected lesions. [^99m^Tc]Tc-MIBI/[^99m^Tc]TcO_4_ scintigraphy was performed in 34 patients and identified 40 suspected lesions. Early acquisitions of [^18^F]FCH PET/CT were performed in 46 patients and identified 52 suspected lesions. Late acquisitions of [^18^F]FCH PET/CT were performed in all 49 patients and identified 52 suspected lesions.

Thirty-one of 49 patients underwent all three examinations (US, [^99m^Tc]Tc-MIBI/[^99m^Tc]TcO_4_ scintigraphy, and [^18^F]FCH PET/CT with both acquisitions), identifying 38 out of 55 lesions.

### 3.2. Surgery and Histopathology Analysis

Histopathology analysis identified 33 (60%) PA, 12 (21.82%) HP, and 2 (3.63%) PC. Eight samples (14.55%) were negative and included normal parathyroid glands (*n* = 2), thyroid tissue (*n* = 2), and reactive lymph nodes (*n* = 4). Immunohistochemistry was carried out in 16 patients, for a total of 19 parathyroid lesions. High expression of Ki-67 was found in eight lesions, including two cases of PC, which presented the highest Ki-67 indices (35% and 40%, respectively). The Ki-67 index in the other 11 cases ranged from 5 to 10%. Furthermore, Ki-67, Gal3, and p53 expression showed an increasing trend from single adenoma to multiple hyperplasia and carcinomas, as shown in Table 2.

### 3.3. Diagnostic Performance of US, [^99m^Tc]Tc-MIBI/[^99m^Tc]TcO_4_ Scintigraphy, and [^18^F]FCH PET/CT

US was performed in 46 patients and correctly identified 33 of 52 lesions (31 TP, 14 FN, 5 FP, and 2 TN), yielding a sensitivity of 68.9%, a specificity of 28.6%, a PPV of 86.1%, a NPV of 12.5%, and an accuracy of 63.5%.

[^99m^Tc]Tc-MIBI/[^99m^Tc]TcO_4_ scintigraphy was performed in 34 patients and correctly identified 25 of 40 lesions (23 TP, 12 FN, 3 FP, and 2 TN), yielding a sensitivity of 65.7%, a specificity of 40%, a PPV of 88.5%, a NPV of 14.3%, and an accuracy of 62.5%.

Early acquisitions of [^18^F]FCH PET/CT were performed in 46 patients and correctly identified 45 of 52 lesions (44 TP, 1 FN, 6 FP, and 1 TN), yielding a sensitivity of 97.8%, a specificity of 14.3%, a PPV of 88.0%, a NPV of 50%, and an accuracy of 86.5%.

Late acquisitions of [^18^F]FCH PET/CT were performed in all 49 patients and correctly identified 46 of 55 lesions (45 TP, 2 FN, 7 FP, and 1 TN), yielding a sensitivity of 95.7%, a specificity of 12.5%, a PPV of 86.5%, a NPV of 33.3%, and an accuracy of 83.6%.

The combination of early and late acquisitions yielded a sensitivity of 97.9%, a specificity of 12.5%, a PPV of 86.8%, a NPV of 50%, and an accuracy of 85.4%.

Overall, [^18^F]FCH PET/CT provided statistically higher sensitivity and accuracy than US and [^99m^Tc]Tc-MIBI/[^99m^Tc]TcO_4_ scintigraphy, without a significant difference between early and late [^18^F]FCH PET/CT acquisitions (*p* = 0.58).

All abnormal [^18^F]FCH uptake foci were identified at both time points, except for one case of HP located in the left inferior position of the thyroid bed, which was not clearly visible on the late scan.

Lesion-based sensitivity, specificity, PPV, NPV, and accuracy of each modality are reported in Table 3.

Per-patient analysis in the whole population (Table 4) and in the subgroup of patients who underwent all three examinations (Table 5) confirmed the statistically higher sensitivity and accuracy of [^18^F]FCH PET/CT compared to US and [^99m^Tc]Tc-MIBI/[^99m^Tc]TcO_4_ scintigraphy.

### 3.4. Measurements of PET Parameters at Early and Late Phases

PA, at both early and late PET/CT acquisitions, showed significantly higher SUVmax compared to normal parathyroids and HP and significantly higher SUVmean compared to HP. PC showed higher LC compared to normal glands at both early and late acquisitions and a higher RI compared to HP (Table 6). Nevertheless, these results derive from only two PC cases, which limits their statistical relevance.

### 3.5. Correlation of PET Parameters with Biochemical Data, Lesion Size, and Histological Analysis

No correlation was found between SUVmax or SUVmean and PTH values. No significant difference was found between any semiquantitative parameters of both early and late PET acquisitions and immunohistochemistry markers (Ki-67, Gal3, BCL2, p53).

A positive correlation was found between late SUVs and calcium levels (Kendall’s tau-b coefficient (tau-b): 0.23, 95% CI: (0.04 to 0.39), *p* = 0.027 for SUVmax; tau-b: 0.20, 95% CI: (0.02 to 0.37), *p* = 0.045 for SUVmean).

Positive correlation was obtained between PET parameters and lesion size values (tau-b: 0.36, 95% CI: (0.18 to 0.51), *p* = 0.0005 for early SUVmax; tau-b: 0.32, 95% CI: (0.13 to 0.48), *p* = 0.002 for early SUVmean; tau-b: 0.37, 95%CI: (0.20 to 0.52), *p* = 0.0002 for late SUVmax; tau-b: 0.35, 95% CI: (0.18 to 0.50), *p* = 0.0004 for late SUVmean late).

Parathyroid lesions were divided into two groups according to their size: ≤1 cm^3^ and >1 cm^3^. SUVmax (early and late) and SUVmean (late) were statistically higher in lesions >1 cm^3^ than in smaller lesions. Late-phase LC was statistically higher in lesions >1 cm^3^ (*p* = 0.04).

Laboratory data and PET parameters according to lesion size are shown in Table 7.

## 4. Discussion

Although the combination of [^99m^Tc]Tc-MIBI/[^99m^Tc]TcO_4_ scintigraphy with US has traditionally been considered the first-line strategy for preoperative localization of hyperfunctioning parathyroid glands in patients with pHPT, the widespread adoption of [^18^F]FCH PET/CT is contributing to changing the diagnostic landscape. 4D-CT is an excellent alternative or complementary examination that could be employed in surgical planning, especially if PET is unavailable [8,10], showing very high diagnostic performance.

In a meta-analysis by Wong et al. [24], [^99m^Tc]Tc-MIBI/[^99m^Tc]TcO_4_ scintigraphy had an estimated pooled sensitivity of 70% (61–80%), 74% (66–82%), and 86% (81–90%) for planar, SPECT, and SPECT/CT, respectively. However, the diagnostic performance of [^99m^Tc]Tc-MIBI/[^99m^Tc]TcO_4_ decreases in multiple parathyroid adenomas and hyperplasia due to the difficulty of localizing small-sized glands [25]. Failure to detect small, intrathyroidal, or ectopic lesions, or adenomas with a rapid [^99m^Tc]-MIBI washout has led to the search for new strategies, particularly using PET radiopharmaceuticals.

Growing evidence from published retrospective and prospective studies, meta-analyses, and randomized controlled clinical trials has demonstrated the higher performance of [^18^F]FCH PET/CT over standard modalities, thus potentially shifting its role to a first-line imaging method [7,12,13,14,18,19,20,21,22].

The present study was designed not only to confirm previous reports, but also to address several other unsolved questions: the optimal scanning time and the biological significance of semiquantitative PET parameters.

In our series, [Tc-99m]Tc-MIBI/[Tc-99m]TcO_4-_ scintigraphy provided 12 FN scans (35.3%). In all these patients, [F-18]FCH PET/CT was TP and achieved 97.8% sensitivity and 86.5% accuracy, in line with previous papers and meta-analyses [12,17,18,26,27,28,29,30,31,32].

Notably, [^18^F]FCH PET/CT performed much better than conventional imaging modalities for the detection of smaller-sized adenomas, multiple-lesion disease, and hyperplasia [27,33]. These differences can be explained by the better contrast between the thyroid gland and the parathyroid adenoma with [^18^F]FCH, and the higher spatial resolution of PET/CT allowing early detection of smaller-sized adenomas [34].

Overall, the higher sensitivity and accuracy, together with lower radiation exposure and shorter acquisition time of [^18^F]FCH PET/CT compared to [^99m^Tc]Tc-MIBI/[^99m^Tc]TcO_4_ scintigraphy, support the use of [^18^F]FCH PET/CT as a first-line imaging technique in suspected pHPT [5,18,19,20,21].

FN results are generally attributed to small lesions below the PET/CT resolution, moderate hypercalcemia, and normal PTH levels. In our study, one and two FN findings on early and late [^18^F]FCH PET, respectively, were observed, corresponding to a small PA located in the right superior site of the thyroid bed, which was also not detected by the first-line imaging techniques.

Since the study population consisted almost entirely of patients with surgically confirmed hyperfunctioning glands, the number of TN lesions/patients was extremely limited, thus allowing high sensitivity but also leading to a low specificity due to a higher number of FP findings in both lesion-based and patient-based analyses.

Consequently, specificity estimates are unstable and associated with very wide confidence intervals and should be interpreted in the context of the study design and patient selection, rather than considered as a definitive estimate of the intrinsic specificity of FCH PET/CT, which has been shown to be considerably higher in larger studies and meta-analyses.

**Figure 2:**
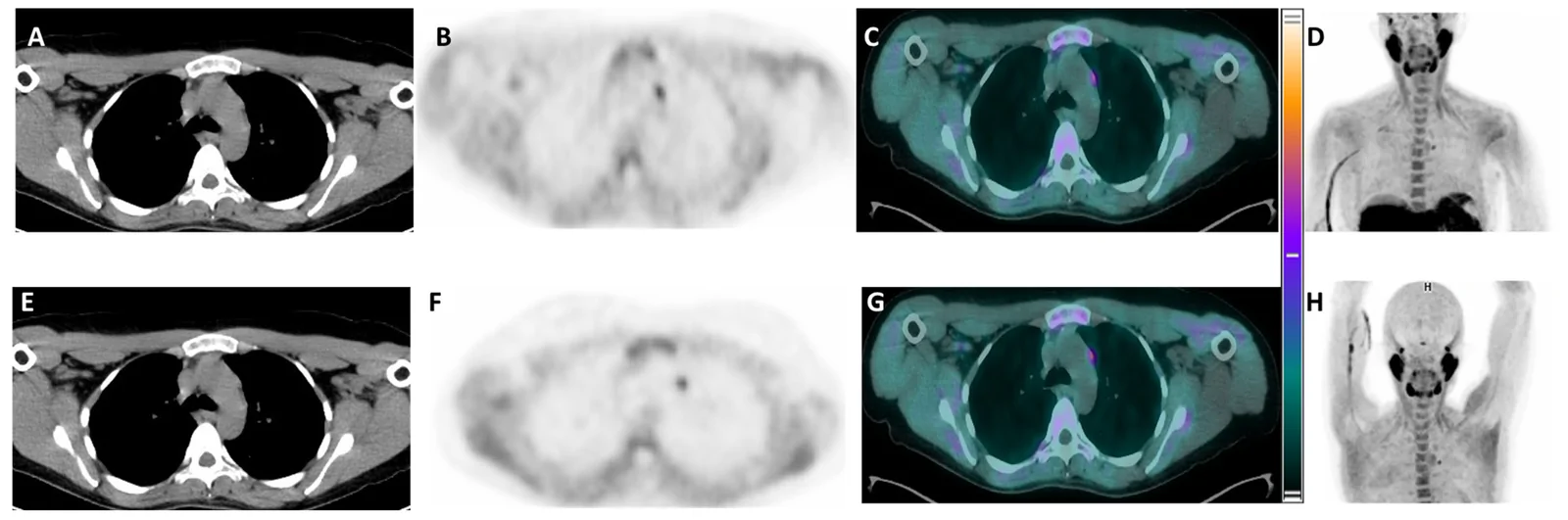
[^18^F]FCH PET/CT of a patient with pHPT and negative first-line imaging. PTH levels at 129.6 ng/L and calcium levels at 11 mg/dL. CT (**A**,**E**), PET (**B**,**F**), PET/CT (**C**,**G**) and maximal intensity projection (**D**,**H**). Images acquired 10 min after [^18^F]FCH injection (**A**–**D**) show a focal uptake of [^18^F]FCH in the anterior mediastinum in proximity to the aortic arch. Images acquired 60 min after injection (**E**–**H**) show an increase in the same focal uptake, suggesting an ectopic hyperfunctioning parathyroid lesion. However, histological analysis after surgery revealed an inflammatory lymph node.

The most commonly reported causes of FP findings are reactive or metastatic lymph nodes (especially in the case of foci in ectopic locations), thyroid nodules, and other benign or malignant tumors [16,18]. For instance, differentiated thyroid cancers are known to mimic hyperplastic parathyroid tissue [1]. In our study, six and seven FP findings were observed on early and late [18F]FCH PET, respectively, corresponding to histologically confirmed lymph nodes, healthy parathyroid glands, or healthy thyroids. Notably, one mediastinal reactive lymph node was wrongly identified as ectopic hyperfunctioning parathyroid tissue due to its high and focal uptake (Figure 2).

Notwithstanding the high and promising diagnostic performance of [^18^F]FCH PET/CT in the detection of hyperfunctioning parathyroid lesions, protocols are variable amongst studies, and no scientific consensus has yet been established regarding the optimal acquisition time.

**Figure 3:**
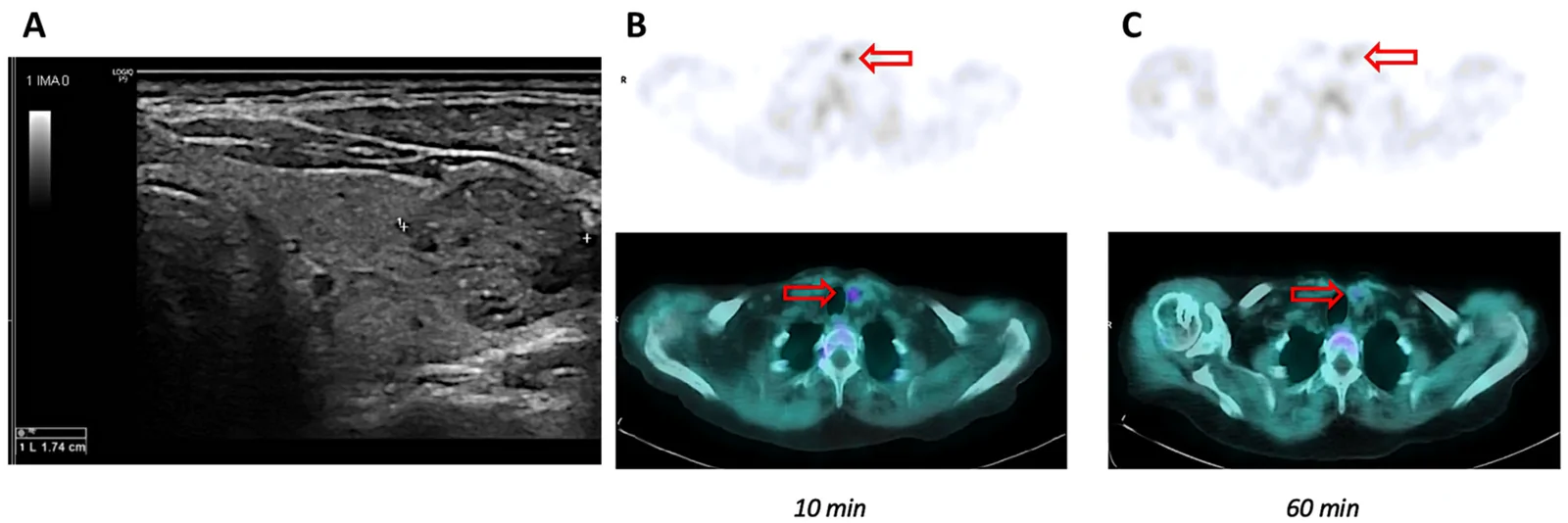
Neck US and [^18^F]FCH of a patient with pHPT (PTH 83.1 ng/L, Calcium 9.3 mg/dL). (**A**) Neck US showed a thyroid nodule in the left lobe but did not detect hyperplastic parathyroid tissue; (**B**) Early-phase images (PET and fused PET/CT) obtained 10 min p.i., showing a focal [^18^F]FCH uptake corresponding to a small nodular lesion located in the left inferior lobe of the thyroid gland (LC = 52.1%). (**C**) Late-phase images (PET and fused PET/CT) obtained 60 min p.i., showing a decreased uptake of the same lesion (RI = −32.87%; LC = 2.1%), indicating a rapid washout of the hyperfunctioning parathyroid lesion (arrow).

Another aim of the present study was to compare qualitatively and quantitatively the PET parameters at 10 min (early phase) and 60 min (late phase) after injection. All TP and TN findings were identified in both the early and late phases, except for one parathyroid lesion with rapid washout (Figure 3).

No significant differences in sensitivity, PPV, and accuracy were found between early and late phases. LC was higher in the late phase than in the early phase, although not significantly. Similarly, Prabhu et al. [34] reported comparable parathyroid/thyroid ratio (P/T ratio) values between early acquisition (15 min) and delayed acquisition (45 min). In contrast with these results, Rep et al. reported a higher LC at late acquisition (one hour and two hours) than at early acquisition (five minutes) [23]. In a study published by Broos et al., the P/T ratio was statistically higher in the late phase (60 min) than at early acquisition (5 min) [35]. These discrepancies with our results may be explained by the choice of performing the early phase at 10 min after injection instead of 5 min, as a decrease in the [^18^F]FCH uptake is observed during the first 10 min after injection, followed by a more stable uptake after 10 min [34,36,37]. Most parathyroid lesions are detectable at both early and late phases. In terms of LC (in relation to the thyroid uptake), the most favorable time point to assess parathyroid lesions appears to be 60 min after injection. However, due to the possible rapid washout of some lesions, a supplementary early acquisition is advisable. The specific timing for this early phase, either at 5 or 10 min after injection, remains unexplored and warrants further investigation.

Our study shows a statistically higher [^18^F]FCH uptake in PA than in HP. However, a threshold using receiver operating characteristic (ROC) curves has not been determined given the small number of HPs. Similarly, two studies reported a higher SUVmax in PA than in HP, but the difference was not statistically significant [13,38]. We found a lower RI in HP compared with PC, indicating washout in the first condition and tracer retention in PC and PA (although no statistically significant difference was found with PA). However, the results of comparisons with PC must be interpreted with caution, as they are based only on two PC cases. Further studies are needed to eventually confirm these preliminary findings.

Similarly, SUVmax and SUVmean (both early and late measurements) may predict the histological nature of a parathyroid lesion, being higher in PA than HP. Determining parameters to distinguish PA from HP could provide crucial information for surgical planning, and studies with larger patient cohorts are needed to confirm whether PET parameters, alone or integrated, can predict the histopathology of a parathyroid lesion.

Calcium levels were positively correlated with [^18^F]FCH uptake in the late phase, and lesion size was positively correlated with [^18^F]FCH uptake at both early and late phases; conversely, no correlation between PTH levels and [^18^F]FCH uptake was observed. Considering that our cohort was small and that only two patients were negative on early [^18^F]FCH PET/CT (one FN and one TN) and three patients were negative on late [^18^F]FCH PET/CT (two FN and one TN), a defined cut-off value for Ca level in order to predict the positivity of [^18^F]FCH PET/CT early or late could not be established rigorously. Piccardo et al. [39] reported similar results. However, other studies show conflicting findings regarding a possible correlation between [^18^F]FCH uptake and biochemical status. Seyedinia et al. reported that SUVmax and SUVmean were correlated with PTH levels, and SUVmax was correlated with calcium levels [40]. Alharbi et al. demonstrated a strong correlation between [^18^F]FCH uptake of PA and PTH level concentration. Of note, amongst the 52 patients included in their study, 42 patients underwent a PET/MR scan, and only 10 patients underwent a PET/CT scan [41]. In a study by Araz et al., PTH levels were statistically higher in patients with high SUVmax [42]. Boccalatte et al. found no correlation between [^18^F]FCH uptake on PET/CT and PTH or calcium levels in patients [43]. Rizzo et al. found that semiquantitative parameters correlated with PTH values, but not with calcium and phosphate levels [44].

Therefore, a reliable correlation between [^18^F]FCH and biochemical parameters is not clearly established and requires further investigation.

In our study, [^18^F]FCH uptake was correlated with parathyroid size measured with US. A positive correlation between [^18^F]FCH uptake and parathyroid lesion size was also observed in studies on [^18^F]FCH PET/MR [45] and [^18^F]FCH PET/CT [39]. However, we noted that some lesions showed an increased uptake related to their size, whereas a subgroup of lesions, particularly parathyroid glands with smaller dimensions, showed an increase in [^18^F]FCH uptake regardless of their size.

Larger, prospective studies are necessary to evaluate the predictive and prognostic role of [^18^F]FCH PET/CT in relation to the histological profile. In our study, the highest Ki-67 indices were observed in the two cases of PC. However, [^18^F]FCH uptake was not correlated with any immunohistochemical marker, including Ki-67. This finding may suggest that [^18^F]FCH uptake is brought about by an abnormal turnover of transmembrane proteins and not by mere cell proliferation, supporting the hypothesis postulated by Ishizuka et al. in 1987, who observed that adenomas presented higher turnover of membrane-associated cyclic AMP-dependent protein kinase C compared to atrophic parathyroid glands, indicating the presence of an association between protein kinase C activity and hypersecretion of PTH [7]. Moreover, Piccardo et al. found a direct correlation between SUV ratio and Ki-67 expression and an inverse correlation between SUV parameters and p53 expression [39]. This information could be of interest as atypical parathyroid adenoma (APA), responsible for 1.2–1.3% of pHPT and presenting malignancy features, demonstrates a higher Ki-67 expression [46,47,48]. Hence, detecting APA with PET parameters could allow closer surveillance of these patients.

This study has several limitations. The single-center and retrospective nature of the study may cause inclusion bias due to our local practice, since [^18^F]FCH PET/CT was performed when US and [^99m^Tc]Tc-MIBI/[^99m^Tc]TcO_4_ scintigraphy was inconclusive. This may have resulted in the inclusion of a selected population and may have overestimated the sensitivity of PET. Nevertheless, this reflects clinical practice and the trend observed in previous years of reserving choline PET for equivocal cases. Nowadays, however, [^18^F]FCH PET is much more widely available, and its diagnostic reliability has been extensively demonstrated; therefore, it is increasingly performed earlier in the diagnostic work-up.

Not all patients underwent all the tests in our institution. Some underwent US and/or [^99m^Tc]Tc-MIBI/[^99m^Tc]TcO4 scintigraphy at another Center. In these few cases, the results were taken into consideration for planning the most appropriate diagnostic and therapeutic approach but were not considered for the calculation of the diagnostic performance of the different imaging modalities. Indeed, in the lesion-based analysis, the patients included in the different imaging modality groups were not necessarily the same, which may limit the direct comparability.

The relatively small sample size of our study does not allow us to draw definitive conclusions, especially regarding the subgroup of 16 patients whose molecular profile was analyzed. Therefore, the ability of SUV parameters and RI to predict the histologic subtype should be interpreted with extreme caution, especially when considering PC, which was diagnosed in only two patients.

4D-CT was not included in the present analysis since it was not routinely performed at the time of the study, although a head-to-head comparison with [^18^F]FCH PET/CT would have been of added value. Moreover, the small number of TN cases, together with the presence of several FP findings, resulted in low and potentially unstable estimates of specificity, which should therefore be interpreted with caution and in the context of the study design and patient selection.

Moreover, we did not include in our analysis the possible added value of new advances in US technology, namely multiparametric US, with the possibility of performing microflow imaging to better characterize small intralesional vessels [49,50], and CEUS, which allows investigation of the different vascular kinetics and contrast enhancement patterns [50], or US elastography, particularly shear wave elastography, which allows assessment of tissue stiffness, thereby providing additional information for differentiating parathyroid lesions from surrounding cervical structures [51]. Unfortunately, these new techniques were not systematically performed at our institution at the time of this study, but they deserve to be compared with other imaging techniques in further studies.

Finally, the performance of a dual-phase [^18^F]FCH PET/CT has already been investigated in the literature, but most of the authors performed the early scan at 5 min after administration of the radiopharmaceutical, earlier than our early phase, which was acquired at 10 min.

Nevertheless, despite these limitations, our findings further support the growing body of evidence demonstrating the value of [18F]FCH PET/CT in the detection of hyperfunctioning parathyroid glands, improving lesion detectability while optimizing healthcare costs and resource utilization [34,35].

## 5. Conclusions

Although the combination of dual-phase dual-tracer [^99m^Tc]Tc-MIBI/[^99m^Tc]TcO_4_ scintigraphy with US is the most widely accepted preoperative strategy for localizing hyperfunctioning parathyroid glands, we confirm the higher sensitivity and accuracy of [^18^F]FCH PET/CT compared with standard first-line modalities. No significant differences in sensitivity, specificity, PPV, NPV, and accuracy were found between early and late acquisitions. The most favorable time point to assess parathyroid lesions appears to be 60 min after injection. However, due to the possible rapid washout of some lesions, a supplementary early acquisition is advisable. The specific timing for this early phase, either at 5 or 10 min after injection, remains unexplored and warrants further investigation. [^18^F]FCH uptake (SUVmax and SUVmean) and the kinetic behavior (represented by the RI) may predict the histological nature of a parathyroid lesion, but this result must be interpreted with caution, as it is based on a limited number of patients. Larger studies are needed to confirm this finding.

## Figures and Tables

**Table 1:** Patient characteristics, laboratory results, and lesion size.

Parameter	Value Mean ± SD (Min to Max)
Gender *	
Female	37 (75.51)
Male	12 (24.49)
Age (years)	56.39 ± 14.24 (26.00 to 80.00)
PTH (ng/L)	365.49 ± 940.40 (63.90 to 6835.00)
Calcium (mg/dL)	11.54 ± 1.87 (5.60 to 19.00)
Lesion size (cm^3^)	2.31 ± 4.08 (0.04 to 17.37)

PTH—Parathyroid hormone; * data presented as *n* (%).

**Table 2:** Analysis of different immunohistochemistry markers in hyperfunctioning parathyroids.

Histology (*n*)	Ki-67		Gal3		BCL2		p53	
	Low	High	Low	High	Low	High	Absent	Present
PA (*n* = 11)	9	2 (18.2%)	10	1 (9.1%)	5	6 (54.5%)	10	1 (9.1%)
HP (*n* = 6)	2	4 (66.7%)	4	2 (33.3%)	2	4 (66.7%)	5	1 (16.7%)
PC (*n* = 2)	0	2 (100%)	0	2 (100%)	2	0 (0%)	0	2 (100%)

Trend of increase in Ki67 *p* = 0.014; for Gal3 *p* = 0.019; for p53 *p* = 0.03.

**Table 3:** Diagnostic performance of neck US, [^99m^Tc]Tc-MIBI/[^99m^Tc]TcO_4_ scan and [^18^F]FCH PET/CT in a lesion-based analysis.

Parameter	A US	B [^99m^Tc]Tc-MIBI/[^99m^Tc]TcO_4_	C Early [^18^F]FCH PET/CT	D Late [^18^F]FCH PET/CT	E Early+ Late [^18^F]FCH PET/CT
Sensitivity (95%CI)	68.9 (55.4 to 82.4)	65.7 (49.9 to 81.4)	97.8 (93.5 to 100)	95.7 (89.9 to 100)	97.9 (93.7 to 100)
Specificity (95%CI)	28.6 (0.0 to 62.0)	40.0 (0.0 to 82.9)	14.3 (0.0 to 40.2)	12.5 (0.0 to 35.4)	12.5 (0.0 to 35.4)
PPV (95%CI)	86.1 (74.8 to 97.4)	88.5 (76.2 to 100)	88.0 (78.9 to 97.0)	86.5 (77.3 to 95.8)	86.8 (77.7 to 95.9)
NPV (95%CI)	12.5 (0.0 to 28.7)	14.3 (0.0 to 32.6)	50.0 (0.0 to 100)	33.3 (0.0 to 86.7.0)	50.0 (0.0 to 100)
Accuracy (95%CI)	63.5 (50.4 to 76.5)	62.5 (47.5 to 77.5)	86.5 (77.3 to 95.8)	83.6 (73.9 to 93.4)	85.4 (76.1 to 94.8)

PPV = Positive Predictive Value; NPV = Negative Predictive Value. Sensitivity—A vs. C, *p* = 0.0002; A vs. D, *p* = 0.0007; A vs. E, *p* = 0.0002; B vs. C, *p* = 0.0001; B vs. D, *p* = 0.0004; B vs. E, *p* = 0.001. Accuracy—A vs. C, *p* = 0.007; A vs. D, *p* = 0.02; A vs. E, *p* = 0.009; B vs. C, *p* = 0.007; B vs. D, *p* = 0.02.

**Table 4:** Per-patient analysis of diagnostic performance of neck US, [^99m^Tc]Tc-MIBI/[^99m^Tc]TcO_4_ scan and [^18^F]FCH PET/CT in the whole population.

Parameter	A US	B [^99m^Tc]Tc-MIBI/[^99m^Tc]TcO_4_	C Early [^18^F]FCH PET/CT	D Late [^18^F]FCH PET/CT
Sensitivity (95%CI)	68.3 (54.0 to 82.5)	67.7 (51.3 to 84.2)	97.6 (92.8 to 100)	95.3 (89.0 to 100)
Specificity (95%CI)	33.0 (0.0 to 71)	66.7 (13.3 to 100)	20.0 (0.0 to 55.1)	16.7 (0.0 to 46.5)
PPV (95%CI)	87.5 (76.0 to 99)	95.4 (86.7 to 100)	90.9 (82.4 to 99.4)	89.1 (80.1 to 98.1)
NPV (95%CI)	13.3 (0.0 to 30.5)	16.7 (0.0 to 37.7)	50.0 (0.0 to 100)	33.3 (0.0 to 86.7)
Accuracy (95%CI)	63.8 (50.1 to 77.6)	67.6 (51.9 to 83.4)	89.1 (80.1 to 98.1)	85.7 (75.9 to 95.5)

PPV—Positive Predictive Value, NPV—Negative Predictive Value. Sensitivity—A vs. C, *p* = 0.0001; A vs. D, *p* = 0.0007; B vs. C, *p* = 0.0006; B vs. D, *p* = 0.002. Accuracy—A vs. C, *p* = 0.004; A vs. D, *p* = 0.01; B vs. C, *p* = 0.02; B vs. D, *p* = 0.049.

**Table 5:** Diagnostic performance of neck US, [^99m^Tc]Tc-MIBI/[^99m^Tc]TcO_4_ scan and [^18^F]FCH PET/CT in 31 patients who performed all three examinations.

Parameter	A US	B [^99m^Tc]Tc-MIBI/[^99m^Tc]TcO_4_	C Early [^18^F]FCH PET/CT	D Late [^18^F]FCH PET/CT
Sensitivity (95%CI)	65.5 (48.2 to 82.8)	65.5 (48.2 to 82.8)	96.5 (89.9 to 100)	93.1 (83.9 to 100)
Specificity (95%CI)	50.0 (0.0 to 100)	100 (100 to 100)	50.0 (0.0 to 100)	50.0 (0.0 to 100)
PPV (95%CI)	95.0 (85.4 to 100)	100 (100 to 100)	96.5 (89.9 to 100)	96.4 (89.5 to 100)
NPV (95%CI)	9.1 (0.0 to 26.1)	16.7 (0.0 to 37.7)	50.0 (0.0 to 100)	33.3 (0.0 to 86.7)
Accuracy (95%CI)	64.5 (47.7 to 81.4)	67.7 (51.3 to 84.2)	93.5 (84.9 to 100)	90.3 (79.9 to 100)

PPV—Positive Predictive Value, NPV—Negative Predictive Value. Sensitivity—A vs. C, *p* = 0.001; A vs. D, *p* = 0.006; B vs. C, *p* = 0.001; B vs. D, *p* = 0.006. Accuracy—A vs. C, *p* = 0.003; A vs. D, *p* = 0.01; B vs. C, *p* = 0.006; B vs. D, *p* = 0.02.

**Table 6:** Summary of calculated semiquantitative parameters.

Parameter	No Parathyroid Disease (A) (*n* = 8) Mean ± SD (95%CI)	HP (B) (*n* = 12) Mean ± SD (95%CI)	PA (C) (*n* = 33) Mean ± SD (95%CI)	PC (D) (*n* = 2) Mean ± SD (95%CI)
SUVmax (early)	4.87 ± 0.98 (3.84 to 5.89)	4.71 ± 2.43 (3.16 to 6.25)	7.17 ± 2.90 (6.09 to 8.25)	6.31 ± 0.31 (3.51 to 9.11)
SUVmean (early)	3.56 ± 0.54 (2.99 to 4.12)	3.24 ± 0.90 (2.67 to 3.81)	3.96 ± 0.80 (3.66 to 4.25)	3.69 ± 0.28 (1.15 to 6.23)
SUVmax (late)	4.81 ± 1.42 (3.50 to 6.12)	4.34 ± 1.77 (3.22 to 5.46)	7.33 ± 3.36 (6.12 to 8.54)	5.95 ± 0.46 (1.82 to 10.07)
SUVmean (late)	3.48 ± 0.37 (3.13 to 3.83)	3.05 ± 0.66 (2.63 to 3.47)	4.03 ± 0.85 (3.72 to 4.33)	3.78 ± 0.07 (3.14 to 4.42)
LC (early)	58.04 ± 113.51 (−61.07 to 177.16)	56.28 ± 61.62 (17.13 to 95.44)	50.38 ± 42.96 (34.33 to 66.42)	21.78 ± 9.33 (−62.09 to 105.65)
LC (late)	102.32 ± 191.01 (−74.34 to 278.98)	61.31 ± 75.89 (13.09 to 109.53)	75.36 ± 66.84 (51.26 to 99.46)	23.53 ± 2.31 (2.77 to 44.29)
RI	−0.29 ± 8.16 (−8.85 to 8.28)	−1.38 ± 28.29 (−19.36 to 16.60)	1.28 ± 8.05 (−1.72 to 4.29)	2.67 ± 5.95 (−50.82 to 56.16)

Post hoc analysis: SUVmax (early): A vs. C, *p* = 0.03; B vs. C, *p* = 0.002. SUVmean (early): B vs. C, *p* = 0.007. SUVmax (late): A vs. C, *p* = 0.01; B vs. C, *p* = 0.0002. SUVmean (late): B vs. C, *p* < 0.0001.

**Table 7:** Laboratory data and PET parameters according to lesion size.

Parameter	Lesion Size ≤ 1 cm^3^ Mean ± SD (95%CI)	Lesion Size > 1 cm^3^ Mean ± SD (95%CI)	*p*
PTH (ng/L)	192.99 ± 186.86 (119.07 to 266.90)	658.51 ± 1491.87 (−39.71 to 1356.72)	0.23
Calcium (mg/dL)	10.95 ± 1.29 (10.42 to 11.48)	12.09 ± 2.26 (10.97 to 13.22)	0.33
SUVmax early	5.54 ± 2.77 (4.47 to 6.62)	7.10 ± 2.51 (5.86 to 8.35)	**0.02**
SUVmean early	3.49 ± 0.79 (3.18 to 3.79)	3.89 ± 0.65 (3.56 to 4.21)	0.08
SUVmax late	5.49 ± 2.93 (4.38 to 6.60)	7.33 ± 3.15 (5.86 to 8.81)	**0.01**
SUVmean late	3.43 ± 0.74 (3.15 to 3.71)	4.00 ± 0.70 (3.68 to 4.33)	**0.009**
LC Early	37.08 ± 38.47 (22.16 to 51.99)	53.13 ± 57.73 (24.42 to 81.84)	**0.017**
LC Late	45.86 ± 50.26 (26.74 to 64.98)	87.24 ± 81.79 (48.97 to 125.52)	**0.048**
RI	0.05 ± 18.04 (−6.94 to 7.05)	2.23 ± 9.75 (−2.62 to 7.08)	0.98

## Data Availability

Some or all datasets generated during and/or analyzed during the current study are not publicly available but are available from the corresponding author on reasonable request.

## Abbreviations

The following abbreviations are used in this manuscript:

pHPT	Primary hyperparathyroidism
PTH	Parathyroid hormone
[^99m^Tc]Tc-MIBI/[^99m^Tc]TcO_4_	[^99m^Tc]Tc-methoxyisobutylisonitrile/[^99m^Tc]Tc-pertechnetate
[^18^F]FCH PET/CT	[^18^F]fluorocholine positron emission tomography computed-tomography
SUVmax	Maximum standardized uptake value
SUVmean	Mean standardized uptake value
RI	Retention index
LC	Lesion contrast
PA	Primary adenomas
HP	Hyperplastic parathyroid gland
PC	Parathyroid carcinoma
